# Glass transition temperature from the chemical structure of conjugated polymers

**DOI:** 10.1038/s41467-020-14656-8

**Published:** 2020-02-14

**Authors:** Renxuan Xie, Albree R. Weisen, Youngmin Lee, Melissa A. Aplan, Abigail M. Fenton, Ashley E. Masucci, Fabian Kempe, Michael Sommer, Christian W. Pester, Ralph H. Colby, Enrique D. Gomez

**Affiliations:** 1 Department of Chemical Engineering, The Pennsylvania State University, University Park, PA 16802 USA; 2 Institute for Chemistry, Chemnitz University of Technology, Strasse der Nationen 62, 09111 Chemnitz, Germany; 3 Department of Materials Science and Engineering, The Pennsylvania State University, University Park, PA 16802 USA; 4 The Materials Research Institute, The Pennsylvania State University, University Park, PA 16802 USA

**Keywords:** Electronic devices, Glasses, Polymers, Rheology

## Abstract

The glass transition temperature (*T*_g_) is a key property that dictates the applicability of conjugated polymers. The *T*_g_ demarks the transition into a brittle glassy state, making its accurate prediction for conjugated polymers crucial for the design of soft, stretchable, or flexible electronics. Here we show that a single adjustable parameter can be used to build a relationship between the *T*_g_ and the molecular structure of 32 semiflexible (mostly conjugated) polymers that differ drastically in aromatic backbone and alkyl side chain chemistry. An effective mobility value, *ζ*, is calculated using an assigned atomic mobility value within each repeat unit. The only adjustable parameter in the calculation of *ζ* is the ratio of mobility between conjugated and non-conjugated atoms. We show that *ζ* correlates strongly to the *T*_g_, and that this simple method predicts the *T*_g_ with a root-mean-square error of 13 °C for conjugated polymers with alkyl side chains.

## Abstract (web summary)

The glass transition temperature (Tg) is a key property that dictates the applicability of conjugated polymers. Here the authors use one adjustable parameter to build a relationship between the Tg and the molecular structure of semiflexible polymers that differ in aromatic backbone and alkyl side chain chemistry.

## Introduction

The glass transition temperature (*T*_g_) of conjugated polymers governs chain dynamics and mechanical properties. Below *T*_g_, the storage moduli of conjugated polymers are on the order of one GPa, making the materials too stiff and too brittle for flexible devices^1^. At higher temperatures, the storage modulus decreases orders of magnitude to ~10 MPa for a semicrystalline polymer, or ~1 MPa for an entangled amorphous polymer^1,2^. Therefore, one of the key design requirements for soft or stretchable electronics is to ensure that *T*_g_ lies below room temperature^3,4^. Furthermore, solution-processing of conjugated polymers for integration into various electronic devices^5–7^ results in morphologies that are far from equilibrium and which tend to evolve with time. The kinetics of morphological evolution with processing or under operation are limited by chain diffusion or segmental motion, which depends on the temperature with respect to *T*_g_. Annealing above *T*_g_ can lead to cold crystallization^8,9^, enhancement of liquid crystalline order^10^, or stronger phase separation with another additive or polymer^11–13^. Thus, *T*_g_ demarks embrittlement and can dictate morphological evolution in the active layer of electronic devices, thereby impacting mechanical and electrical performance.

Quantitatively predicting the glass transition temperature of conjugated polymers from the chemical structure remains a challenge. *T*_g_ appears to increase with both chain stiffness and bulkier side groups^14–17^, and decreases as alkyl side group length increases^1^, but universal molecular models that connect the *T*_g_ to a given repeat unit structure are lacking. Nevertheless, a connection between the glass transition and the local motion of atoms has been proposed. Measures of the Debye–Waller factor 〈*u*^2^〉 describe local atomic displacements and define a characteristic volume of motion^18^, the dynamic free volume (*v*_f_)^19–24^. *T*_g_ has been successfully predicted from either the structural relaxation time, which is found to be universally related to 〈*u*^2^〉, or *v*_f_, using the generalized Lindemann criteria^15,19,25^. Further connecting local motions to complex chemical structures could extend the predictive power of these models.

Other approaches have used data to build empirical correlations based on the quantitative structure-property relationships (QSPR) method^26^, group contributions approaches^27^, and machine learning^28^ to predict and match with experimentally measured *T*_g_s of many polymers. For QSPR, each bond in the repeat unit is assigned a bond flexibility value, which is further weighted by various molecular descriptors that capture the atomic variance in mass and polarizability^29–31^. Then, a multistep linear regression analysis that trains these descriptors eventually allows a good match between predicted and experimentally measured *T*_g_ values, but also generates many parameters that are challenging to physically interpret, such as topological bond connectivity values^26,31^. Similarly, group contribution methods empirically assume that structural groups in the repeat unit provide weighted additive contributions to the *T*_g_, and that the contribution from one group also depends on the presence of other nearby groups^27^. Using a large data set of 854 polymers, while considering 229 parameters (i.e., descriptors), a model composed of 69 dimensions can predict the *T*_g_ with a root-mean-square error of 24 °C^28^. In all of the aforementioned cases, these methods require at least five parameters to predict the *T*_g_ from the chemical structure. We thus propose that a simpler method, similar to the seminal work by Boyer^32^ and Van Krevelen^27^ on flexible polymers, is warranted for the design of conjugated polymers, given the large number of different backbones and side groups that are possible.

The *T*_g_ of conjugated polymers is notoriously difficult to identify, because many such materials are semicrystalline and have rigid backbones. Signatures of *T*_g_ in differential scanning calorimetry (DSC) scans are thereby suppressed^33^, although some unambiguous values have been summarized by Müller^34^. Alternatively, rheological measurements are sensitive to changes in mechanical properties, and can unambiguously locate the *T*_g_ of conjugated polymers as the peak in the loss modulus as a function of temperature^8^.

**Fig. 1:**
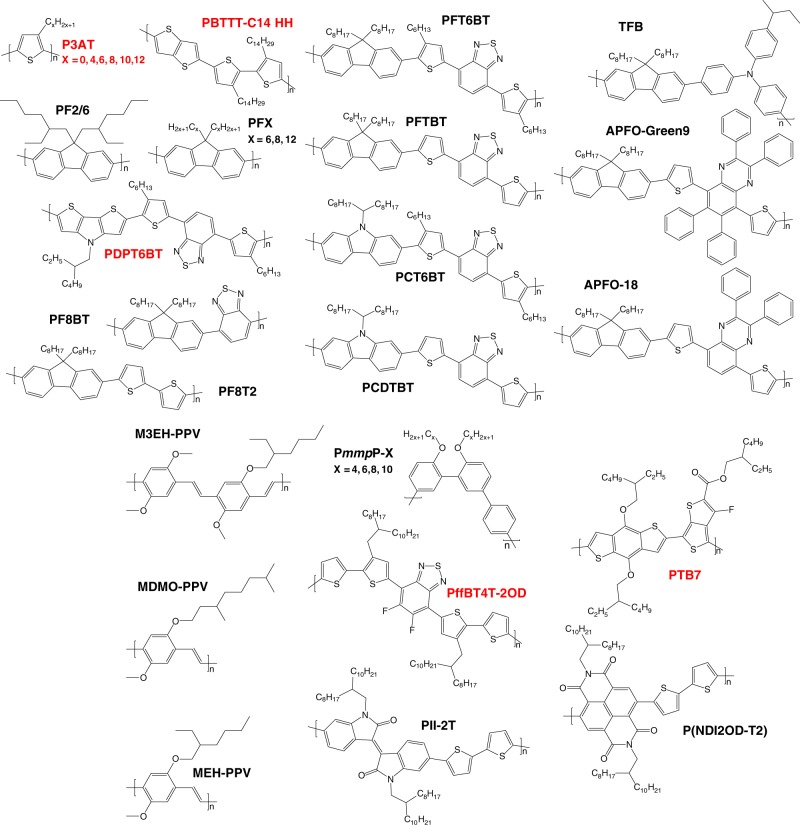
Chemical structures of polymers with aromatic backbones and aliphatic side chains used in this work. Polymers that are highlighted in red belong to Group 1 (mostly thiophene-rich polymers), others belong to Group 2 (mostly phenyl-rich polymers). Full names are found in Supplementary Note 1.

In this work, we obtained glass transition temperatures using rheology of various polymers, with values between −50 °C and 200 °C. We use *T*_g_s from rheology to derive a simple correlation between *T*_g_ and the chemical structure of conjugated polymers with alkyl side chains. Altogether, we examine materials based on polyalkylthiophene, polydialkylfluorene, polyarylene, poly(*p*-phenylene vinylene), and donor-acceptor alternating architectures (or “push-pull” polymers), including macromolecules with linear and branched side chains. We find that an effective atomic mobility parameter is strongly correlated with *T*_g_ for all 32 polymers included in this study. The chemical structures of the various types of semiflexible polymers with different alkyl side chains are shown in Fig. 1, and their full chemical names and sources are summarized in Supplementary Note 1. We demonstrate that our universal description holds for both conjugated and non-conjugated polymers with aromatic backbones. Thus, the correlation between atomic mobility and *T*_g_ is rationalized to result from the common structural scheme of rigid aromatic backbones and flexible alkyl side chains. We expect this correlation between *T*_g_ and the effective atomic mobility to guide the design of conjugated polymers and other rigid aromatic polymers for future applications, including soft, flexible, and stretchable electronics.

## Results

### Measurements of glass transition temperatures

Given the rich phase behavior of conjugated polymers, dynamic mechanical analysis, such as linear shear rheometry, is a reliable and unambiguous way to identify the glass transition temperature^3,8,34^. Because of the dynamic nature of the glass transition response, *T*_g_ is known to be highly dependent on the probing parameters, such as the rate of heating and the frequency of mechanical oscillation. Therefore, in order to consistently compare *T*_g_s of various conjugated polymers in this work, *T*_g_ is determined by either rheology with a heating rate of 5 °C/min and an oscillation frequency of 1 rad/s, or with differential scanning calorimetry (DSC) using a heating rate of 10 °C/min, where *T*_g_ is taken as the midpoint of the transition.

**Fig. 2:**
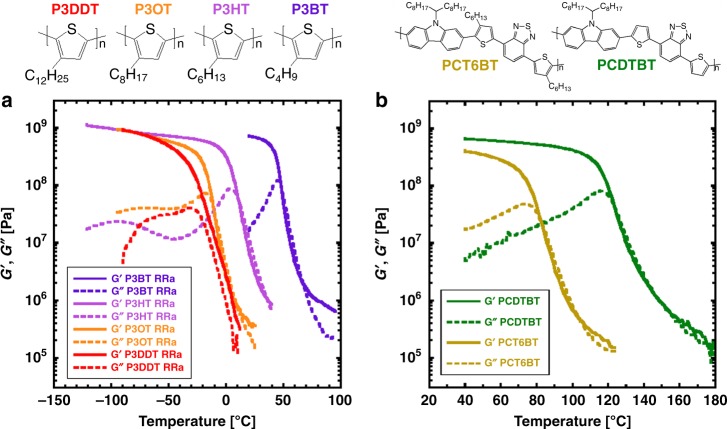
Locating the glass transition temperature using rheology. **a** Storage (*G*′) and loss (*G*″) moduli for regiorandom P3ATs with different side chain lengths (P3BT, P3HT, P3OT, and P3DDT). **b** *G*′ and *G*″ for PCDTBT and PCT6BT. Strain amplitude of 0.001, frequency of 1.0 rad/s, heating rate of 5 °C/min. The glass transition temperature is taken at the peak in *G*″.

Specifically, *T*_g_ is located as the temperature that shows either the local maximum of *G*″ in Fig. 2, Supplementary Figs. 1–4, or a step change in the heat flow in Supplementary Figs. 5 and 6. In Fig. 2a, the local maximum of *G*″ at lower temperatures has been attributed to alkyl side chain relaxation, while the local maximum of *G*″ at higher temperatures is associated with the segmental backbone relaxation; this classification was verified by studying the dependence of interlayer spacing on the side chain length of P3ATs^16,35^. As the side chain length of poly(3-alkylthiophene) (P3AT) increases, the glass transition temperature of the backbone shifts to a lower temperature while that of the side chain increases. These changes in backbone and side chain *T*_g_s have been observed previously and are attributed to “internal plasticization” of the backbone by the highly mobile alkyl side chains^16^. Not all conjugated polymers exhibit two *T*_g_s, such as poly((9,9-dioctylfluorene)-2,7-diyl-alt-[4,7-bis(thiophen-5-yl)-2,1,3-benzothiadiazole]-2′,2″-diyl) (PFTBT)^8^. We speculate that side chain nanodomains for PFTBT are not large enough to exhibit a measurable side chain *T*_g_ due to the smaller side chain fraction than in P3ATs^8^. Nevertheless, backbone *T*_g_s for other push-pull polymers, such as poly[N-9′-heptadecanyl-2,7-carbazole-alt-5,5-(4′,7′-di-2-thienyl-2′,1′,3′-benzothiadiazole)] (PCDTBT), also show an ~50 °C decrease when hexyl side chains are added to the thiophene units (Fig. 2b).

On the other hand, for a set of conjugated polymers with the same branched side chains but different backbones, the side chain *T*_g_ is found to be roughly the same while the backbone *T*_g_ changes appreciably (see Supplementary Fig. 4 and Supplementary Note 2). Additionally, the *G*″ peak corresponding to the glass transition for these branched side chains appears sharper than that of the backbone in Supplementary Fig. 4, in contrast to the case of regiorandom P3ATs in Fig. 2a. Clearly, *T*_g_ depends on the length and number of alkyl side chains attached to the conjugated backbone. *T*_g_ also depends modestly on the molecular weight, by ~15 °C for molecular weights between 10 and 100 kg/mol^8^.

### Correlation between *T*_g_ and side chain mass fraction

**Fig. 3:**
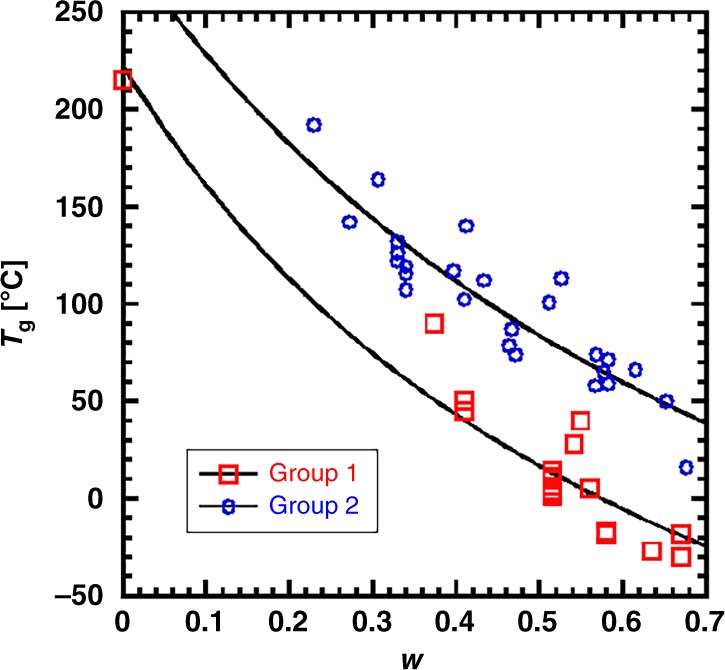
Correlation between the side chain mass fraction (*w*) and the glass transition temperature (*T*_g_) for conjugated polymers in this work. Two groups, representing thiophene-rich polymers (Group 1, red squares) and phenyl-rich polymers (Group 2, blue circles), are modeled using Eq. (1) with fitting parameters shown in Table 1.

We now focus on the *T*_g_ that first demarks embrittlement upon cooling, the backbone glass transition. We first show the dependence of the backbone *T*_g_ on the alkyl side chain mass fraction, *w*. The value for *w* is directly calculated from the repeat unit structure (ignoring chain ends) in Supplementary Note 3 and is summarized in Supplementary Table 1 along with the corresponding *T*_g_ values. Figure 3 shows *T*_g_ versus *w*, where data can be roughly classified into two groups: structures in Group 1 are rich with thiophene rings; Group 2 structures are dominated by phenyl rings. This assignment is imperfect, but highlights potential insights. For instance, the trends for both Group 1 and Group 2 agree with many other polymer systems that have stiff backbones and increasing lengths of flexible side chains^16,36,37^. The suppression of *T*_g_ due to internal plasticization from flexible side chains is expected, as has been demonstrated from a generic monomer model that predicts the *T*_g_^15^. In order to better quantify the difference between the two Groups in Fig. 3, the data were modeled with the Fox equation^38^,

1 $$\frac{1}{{T_{\mathrm{g}}}} = \frac{w}{{T_{{\mathrm{g,sc}}}}} + \frac{{1 - w}}{{T_{{\mathrm{g,bb}}}}}$$

by treating these polymers as nanophase separated mixtures of alkyl side chains (subscript sc) and conjugated backbones (subscript bb) with corresponding glass transition temperatures for each as *T*_g,sc_ and *T*_g,bb_.

As shown in Table 1, both *T*_g,sc_ and *T*_g,bb_ differ significantly between Groups 1 and 2, suggesting that the side chain mass fraction alone cannot fully account for differences in the glass transition temperature of conjugated polymers. In particular, phenyl-rich backbones have a 69 K higher *T*_g,bb_ than thiophene-rich backbones even in the limit of no side chains, because different rings contribute differently to *T*_g_, as suggested in predictions made by Dudowicz^15^ and experiments by Sokolov^39^. This finding motivates refined models connecting the chemical structure and *T*_g_, as discussed below.

**Table 1:** Fitting parameters of the Fox Equation used to describe the *T*_g_ of Groups 1 and 2.

Group	*T*_g,sc_ [°C]	*T*_g,bb_ [°C]
Group 1 (thiophene-rich)	−69 ± 4	218 ± 13
Group 2 (phenyl-rich)	−14 ± 8	287 ± 25

### Correlation between *T*_g_ and packing length

Conjugated polymers have a common structural scheme, a comb-like architecture with a stiff conjugated backbone and “flexible” alkyl side chains. Although more flexible than the backbone, alkyl side chains are rather stiff, given that a dodecyl side chain is about one Kuhn length of polyethylene^40^. Nevertheless, based on the general trend that the backbone *T*_g_ is higher for stiffer polymers with shorter alkyl side chains, the persistence length (*l*_p_) and the Kuhn monomer volume might be key dimensional parameters that govern the *T*_g_s of conjugated polymers (see Supplementary Note 4). *l*_p_ is determined by either light scattering or the freely rotating chain model as shown in Supplementary Fig. 7. The packing length (*p*), which is a ratio of the Kuhn monomer volume over the square of the Kuhn length (2*l*_p_), is then a natural candidate to be correlated with *T*_g_. Nevertheless, as shown in Supplementary Fig. 8, the *T*_g_s of conjugated polymers do not follow a universal relationship with the packing length or other physical chain dimensions, such as persistence length, Kuhn monomer volume, or chain thickness. Instead, we speculate that the dynamic free volume swept out by atoms on short times scales are correlated with chain and segmental motions that dictate the glass transition, which has a timescale on order of 100 s^19,24,41,42^.

### Correlation between *T*_g_ and effective atomic mobility

The rough partitioning of data shown in Fig. 3 into two groups based on two predominant moieties suggests that the value for the *T*_g_s of conjugated polymers may be described as an interpolation between *T*_g_s of the flexible side chains and that of conjugated cores. We thus propose a semi-empirical correlation between the backbone *T*_g_ and an effective mobility of the repeat unit, *ζ*, which captures the local mobility or stiffness averaged over the constituent side chain and backbone structure. We calculate *ζ* by summing assigned atomic mobilities *ζ*_i_ while excluding hydrogens (i.e., akin to the united atom model), and while ignoring the atomic heterogeneity between carbon, nitrogen, sulfur, and oxygen. Each atom that is part of a side chain (either C–C or C–O single bond) is assigned a mobility of 1, while each atom that is part of an aromatic ring or the backbone is assigned a value *a* (*a* < 1). Thus, we normalize the mobility of each atom to that of an atom within a flexible chain. We specify that each ring, thiophene or phenyl, contributes equally to the *T*_g_ by constraining *ζ*_i_ per atom with Eq. (2):

2 $${\mathrm{6}} \times \zeta _{{\mathrm{phenyl}}} = {\mathrm{5}} \times \zeta _{{\mathrm{thiophene}}}$$

This contribution of aromatic rings to the *T*_g_ with respect to the contribution of flexible side chains is a fitting parameter for our model. Assigning atoms within phenyl rings a value of *a* then sets values for atoms within thiophene rings at 1.2*a*, making the total contribution of either ring to *ζ* to be 6*a*. Other atoms on the backbone that are not part of an aromatic ring (e.g., alkenyl and carbonyl groups) are assigned values of *a*, or the same as atoms within phenyl rings.

Using the assignments summarized in Table 2, the mobility of the repeat unit *ζ* is calculated by summing the mobilities of the constituent atoms while normalizing by the total number of atoms (excluding hydrogens) in the entire repeat unit according to Eq. (3) below:

3 $$\zeta = \frac{{{\sum} {\zeta _i} N_i}}{{{\sum} {N_i} }} = \frac{{\zeta _{{\mathrm{phenyl}}}N_{{\mathrm{phenyl}}} + \zeta _{{\mathrm{thiophene}}}N_{{\mathrm{thiophene}}} \pm \zeta _{{\mathrm{dbl}}\,{\mathrm{bond}}}N_{{\mathrm{dbl}}\,{\mathrm{bond}}} + \zeta _{{\mathrm{flexible}}}N_{{\mathrm{flexible}}}}}{{N_{{\mathrm{phenyl}}} + N_{{\mathrm{thiophene}}} \pm N_{{\mathrm{dbl}}\,{\mathrm{bond}}} + N_{{\mathrm{flexible}}}}}$$

where *N*_i_ is the number of atoms (excluding hydrogens) in the repeat unit belonging to a phenyl ring (*N*_phenyl_), a thiophene ring (*N*_thiophene_), an alkenyl or carbonyl group (*N*_dbl bond_), and a side chain (*N*_flexible_), while *ζ*_i_ represents the effective atomic mobility for the corresponding unit. Contributions of atoms linked by double bonds are added to account for weakly mobile atoms on the backbone that are not part of aromatic rings, such as the vinylene moiety in PPVs and carbonyl groups in P(NDI2OD-T2), PII-2T, and PTB7. We account for fused rings by considering them as two rings minus the missing number of atoms with mobilities of *a* that are needed to complete two independent rings, thereby subtracting an *ζ*_dbl bond_*N*_dbl bond_ term to avoid double-counting. For instance, thienothiophene in PBTTT-C14 and benzodiathiazole in PFTBT can be treated as sums of two conjugated rings minus two conjugated carbons (12*a* – 2*a* = 10*a*). As a consequence, calculating *ζ* from the chemical structure of any conjugated polymer depends on a single parameter *a*, which describes the dynamics of the conjugated core with respect to the side chains.

**Table 2:** Assignment of effective atomic mobility value for each atom in different units, namely phenyl rings, thiophene rings, alkenyl or carbonyl groups, flexible C–C or C–O side chains.

**Fig. 4:**
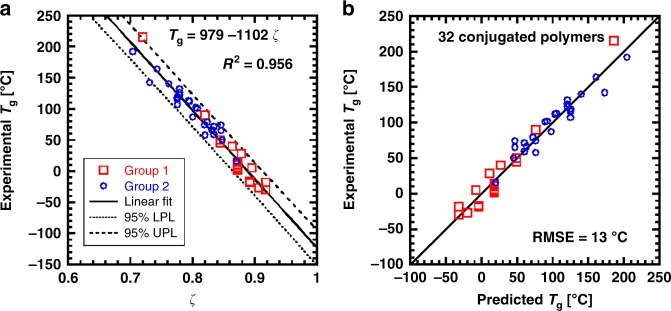
Predicting glass transition temperature from effective mobility value. **a** General correlation between the experimentally measured glass transition temperature (*T*_g_) and the effective mobility value (*ζ*) for conjugated polymers with different backbones and alkyl side chains. Solid line represents the best linear fit through all data with the coefficient of determination (*R*^2^) shown above. Dotted and dashed lines represent the 95% lower and upper prediction line (LPL and UPL), respectively. Group 1 (red squares) is composed of thiophene-rich backbones and Group 2 (blue circles) is composed of phenyl-rich backbones, as introduced in Fig. 3. **b** Comparison between experimental *T*_g_ and prediction by Eq. (4) for all 32 alkylated conjugated polymers, which yields a root-mean-square error (RMSE) of 13 °C.

As shown in Supplementary Note 5, setting *a* = 0.60 in Supplementary Fig. 9 maximizes the linear correlation between *T*_g_ and *ζ* for the 32 polymers examined in this work. Relaxing the constraint on the same mobility per ring and introducing another adjustable parameter for the atomic mobility in a thiophene ring (different than that in a phenyl ring) does not significantly improve the strength of correlation between *T*_g_ and *ζ*. Therefore, we uphold our original assignment of the atomic mobility in Table 2, confirming the use of a single parameter *a* in the calculation of *ζ* from the repeat unit. The effective mobility values for all 32 aromatic polymers in this study are summarized in Supplementary Table 1. Despite the drastic differences in chemical structure, Fig. 4a shows that larger values of *ζ*, which correspond to more mobile polymers, have lower *T*_g_, such that

4 $$T_{\mathrm{g}} = {\mathrm{979}} - {\mathrm{1102}}\,\zeta \,^\circ {\mathrm{C}}$$

95% confidence intervals for predictions based on Eq. (4) are also shown in Fig. 4a as dotted and dashed lines. Additionally, the direct comparison between the predicted *T*_g_ and the experimental *T*_g_ is shown in Fig. 4b, with a root-mean-square error (RMSE) of 13 °C. In the limit that all atoms are completely mobile (i.e., *ζ* = 1), this correlation in Fig. 4a predicts a *T*_g_ of −120 °C, which is comparable with the *T*_g_ of polyethylene^43–46^ and the alkyl side chain *T*_g_s of P3HT (−100 °C), P3OT (−65 °C), and P3DDT (−50 °C)^16^. In the other limit, that of conjugated polymers without any side chains, the *T*_g_ of unsubstituted polythiophene (*ζ* = 0.72) lies reasonably close to the correlation, even though this value is determined from a weak signature in DSC (Supplementary Fig. 6). No signatures of *T*_g_ are detected for poly(*p*-phenylene) before degradation. Nevertheless, our model is consistent with the extrapolations to zero side chains shown in Fig. 2 and Table 1 (*T*_g,bb_) for polyphenylene vinylene (PPV, *ζ* = 0.6, predicted *T*_g_ = 318 °C) and for polythiophene (*ζ* = 0.72, predicted *T*_g_ = 186 °C).

## Discussion

Given the success of our simple relationship between chemical structure and *T*_g_, we surmise that the glass transition in these materials is mainly governed by the local dynamics of the repeat unit. For instance, although the kinked polyarylenes (PAr) have much more flexible backbone conformations than polyfluorenes and other push-pull polymers (persistence lengths of 1 nm versus ~7 nm), both P*mmp*P-8 and PF6 show similar effective mobility values of *ζ* ~0.80 and similar observed *T*_g_s at around (94 ± 7) °C. Thus, polymers with “stiffer” backbones do not necessarily have higher *T*_g_s. Furthermore, because evidence for two *T*_g_s for all conjugated polymers is not always observed, we hypothesize that the success of our correlation in Fig. 4 could imply two possibilities. Perhaps microphase separation of the side chains does not affect the backbone *T*_g_, due to a decoupling of segmental dynamics between the backbone and side chains even without microphase separation. Alternatively, all conjugated polymers studied here could exhibit microphase separation of the side chains, but perhaps not all side chain *T*_g_s are detectable with our current metrology. In addition, the length or branching of the side chain is not directly related to the backbone *T*_g_ within the accuracy of our model, and instead contributes by varying the amount of mobile atoms. Figure 4 also includes both conjugated and non-conjugated backbones (PAr’s), altogether thereby demonstrating a powerful approach to predict the *T*_g_ of aromatic polymers.

Our correlation includes *T*_g_s for P3HTs (both regioregular and regiorandom), PFTBTs and PCDTBTs that vary in molecular weight, and as a consequence exhibit *T*_g_s that differ by about 15 K at most for the same polymer; these are still within the 95% prediction band (i.e., about ±26 K in this case). But, as the molecular weight (see Supplementary Note 1) becomes smaller or close to its Kuhn monomer molar mass, the packing of these short and stiff chains would be drastically different than that of a Gaussian coil in the long chain limit, which may lead to a larger deviation beyond the prediction band of our current model. As shown in Supplementary Table 1, the molecular weight of every polymer is larger than its corresponding Kuhn monomer molar mass, thereby ensuring a modest effect of molecular weight on *T*_g_.; even when the number of Kuhn segments approaches 1, predicted values are quite accurate. In addition, crystallization can perturb the *T*_g_ by perturbing chain mobilities near crystal interfaces or chain conformations in the amorphous phase; this effect is again on the order of about 15 K for conjugated polymers^8^. Thus, using *T*_g_s that are extrapolated to infinite molecular weight, and, if necessary, extrapolated to zero crystallinity, could further improve the accuracy of our model.

We also consider the case of phenyl rings at the end of side chains, which are not as common as alkyl moieties (see Supplementary Note 6). As shown in Supplementary Fig. 10a, data for polystyrene (PS) and poly[2-methoxy-5-(2′-phenylethoxy)-1,4-phenylenevinylene] (MPE-PPV) lie below the correlation. This suggests that the atoms in the terminal phenyl ring are much more mobile than those of phenyl rings on the backbone, thus increasing the average value of the atomic mobility in a phenyl ring at the end of a side chain (*ζ*_phenyl,side chain_). Indeed, by increasing *ζ*_phenyl, side chain_ from 0.60 to 0.72, both PS and MPE-PPV collapse with the rest of the data (Supplementary Fig. 10b). Along these lines, APFO-18, which has two singly attached phenyl rings as side groups, is right on the dotted lower prediction line of Fig. 4.

Although the assignment of the atomic mobility in an aromatic ring with respect to the mobility of alkyl side chains is obtained as an empirical parameter, there is significant evidence for a connection between atomic motion and *T*_g_. One interpretation of our atomic mobility *ζ* is as the average local stiffness, which has been predicted to be related to short timescale motion, the dynamic free volume, and as a consequence the *T*_g_^19,47^. We can use MD simulations to compare the motion of the conjugated core and carbon atoms on the side chain next to the core, and thereby minimize the contributions of long timescale diffusion to the motion of side chains. Previous work on amorphous P3HT and P3DDT at 300 K show that the ratios of the root-mean-square displacement within 2 ns, at the thiophene ring versus the closest atom on the side chain, are 0.69 for P3HT and 0.77 for P3DDT^48^, which is close to the value used for the correlation shown in Fig. 4 (*ζ*_thiophene_/*ζ*_flexible_ = 1.2*a* = 0.72, where *a* = 0.6).

The connection between atomic mobility and the *T*_g_, perhaps through local motion, implies some limits to our correlation shown in Eq. (4) and Fig. 4. For example, using non-alkyl side chains, such as those based on siloxane, could lead to different backbone-to-side chain atomic mobility ratios. Adding flexible alkyl segments to conjugated backbones or adding stiff rings to alkyl side chains (as shown for PS and MPE-PPV) should warrant different values of *a* for these moieties.

In summary, we propose a simple approach to predict the glass transition temperature from the chemical structure of conjugated polymers using a single adjustable parameter. Our correlation between the average mobility of constituent atoms and *T*_g_ provides an estimate that is accurate over a wide variety of rigid, aromatic (co)polymers. The side chain mass fraction and the packing length are only correlated to *T*_g_ for a limited group of conjugated polymers with either a particular backbone unit or a specific range of chain stiffness. But, a general correlation between the *T*_g_ and the effective mobility value of the repeat unit, *ζ*, is observed for all 32 polymers in this work, with 0.7 ≤ *ζ* ≤ 0.92 showing −30 °C ≤ *T*_g_ ≤ 215 °C. In essence, *ζ* represents the effective number of mobile atoms in a repeat unit based on the assigned mobility values for atoms *ζ*_i_ belonging to different units, including a phenyl ring, an alkenyl or a carbonyl group (*ζ*_i_ = 0.60), a thiophene ring (0.72) on the backbone, and an alkyl group (1.0) on the side chain. As a result, this correlation between the backbone *T*_g_ and *ζ* predicts the *T*_g_ with a root-mean-square error of 13 °C.

If we assume the target backbone *T*_g_ for stretchable, electrically-active polymers that operate around room temperature to be ~0 °C or lower, our results imply an effective mobility value *ζ* of 0.89 or larger. This roughly requires at least one octyl side chain per thiophene ring or one hexadecyl side chain per phenyl ring. But, side chain crystallization is likely to occur for dodecyl or longer linear side chains, thus inhibiting stretchability. Nevertheless, our analysis shows that not every ring needs to be alkylated as in the poly(3-alkylthiophene-2,5-diyl) family, but instead side chains can be part of long branched groups on selected monomers. This architecture provides opportunities for strong π-π stacking, and could thereby promote intermolecular delocalization in materials that are rubbery at room temperature.

## Methods

### Materials

As denoted in the Supplementary Note 1, many of the polymers examined here were purchased from commercial sources, such as the polyalkylthiophenes with different side chain lengths (PT, P3BT, P3HT, P3OT, P3DT, and P3DDT), polydialkylfluorenes with different side chain lengths (PF6, PF8, and PF12), low bandgap polymers with branched side chains (PffBT4T-2OD, PTB7, and P(NDI2OD-T2)), and some donor-acceptor alternating copolymers (PF8BT and PF8T2). In addition, various alternating copolymers were synthesized, including PFTBT, PCDTBT, PFT6BT, PCT6BT, PDPT6BT^49^, and PII-2T^50^, using previously reported procedures. The side chains of PBTTT-C14 HH are intentionally designed with head-to-head arrangement to distort the backbone planarity and suppress the crystallization of backbones, thus allowing a more apparent glass transition response. 5,5ʹ-dibromo-3,3ʹ-ditetradecyl-2,2ʹ-dithiophene for PBTTT-C14 HH was prepared by the Grignard reaction of 2-bromo-3-tetradecyl thiophene followed by bromination. Two equivalents of 2-bromo-3-tetradecyl was reacted by using active magnesium solution in THF (purchased from Rieke Metals, LLC) and [1,3-bis(diphenylphosphino)propane]dichloronickel(II) (Ni(dppp)Cl_2_) (purchased from Sigma-Aldrich, Inc.) as catalyst. Unreacted 2-bromo-3-tetradecyl thiophene was removed by vacuum distillation. The product passed through a short column with hexane for further purification. For bromination, 2.2 equivalents of *N*-bromosuccinimide (NBS) was added by 8 portions into 3,3ʹ-ditetradecyl-2,2ʹ-dithiophene solution in chloroform/acetic acid 50:50 (v/v). PBTTT-C14 HH was prepared through Stille reaction of 5,5ʹ-dibromo-3,3ʹ-ditetradecyl-2,2ʹ-dithiophene and 2,5-Bis(trimethylstannyl)thieno[3,2-b]thiophene (purchased from Sunatech, Inc.) in toluene by using tris(dibenzylideneacetone)dipalladium(0) (Pd_2_(dba)_3_) and tri(*o*-tolyl)phosphine. After polymerization at 90 °C for 48 h, the product was precipitated in methanol, followed by Soxhlet extraction with methanol and acetone subsequently. Polyarylenes with different side chains (P*mmp*P-4, P*mmp*P-6, P*mmp*P-8, and P*mmp*P-10) were synthesized as previously described^51^. Chemical structures of conjugated polymers with clearly reported *T*_*g*_s but not measured here are shown in Fig. 1 as well, including poly(*p*-phenylene vinylene)s (M3EH-PPV, MDMO-PPV, and MEH-PPV), TFB, PF2/6, APFO-Green9, and APFO-18. Also included are the *T*_*g*_s of multiple molecular weights of PFTBT, PCDTBT, and regioregular (RR) and regiorandom (RRa) P3HT from our previous work^8^. Molecular weights were determined as discussed in Supplementary Note 7.

### Measurement of *T*_g_ by rheology and DSC

*T*_g_s are identified using linear viscoelastic rheology, except for the completely amorphous polymers (PAs) that show clear glass transition signatures in DSC experiments and an unmoldable polymer (PT) where DSC is the only option to probe its *T*_g_. Samples for rheology are molded under vacuum in a nitrogen-filled glove box at a temperature well above their highest reversible thermal transitions. Only ~15 mg of sample is needed to form a puck with 3 mm diameter and roughly 1 mm thickness. The mass density is estimated by weighing the puck and measuring its thickness in the rheometer as summarized in Supplementary Table 1. Rheology measurement is then carried out in a strain-controlled rotational rheometer (TA Instruments ARES-G2) under a nitrogen environment. The molded puck is loaded in the rheometer between two 3 mm-diameter aluminum parallel plates at room temperature and then annealed at 20 °C above the melting temperature or 100 °C above *T*_g_ to ensure good adhesion with the plates.

Because PTB7 does not melt below 320 °C, we determine this *T*_g_ by gluing a puck molded at 300 °C onto the rheometer plates using cyanoacrylate; this glue limits experiments to below 100 °C. In this work, *T*_g_ is located as the temperature that shows the local maximum of the loss modulus *G*″ during a heating scan with a rate of 5 °C/min, a frequency of 1 rad/s, and an oscillatory strain amplitude of 0.001. For conjugated polymers that exhibit two *G*″ peaks when *G*′ decreases starting on the order of 1 GPa, the higher temperature transition is labeled as the *T*_g_ of the backbone, while the lower temperature to that of the side chain. Specific examples are highlighted in the Results section to support this assignment. In this work, all *T*_g_s refer to the backbone glass transition temperatures unless otherwise stated.

## Supplementary information

Supplementary Information

## Data Availability

The authors declare that the data that support the findings of this manuscript can be found in the Supplementary Information and are available free of charge on Penn State’s ScholarSphere at https://scholarsphere.psu.edu/collections/prr171z013.
