# Supplementary material for: Glass transition temperature from the chemical structure of conjugated polymers
Source: Nat Commun. 2020 Feb 14;11:893. doi: 10.1038/s41467-020-14656-8 (PMC7021822; doi:10.1038/s41467-020-14656-8)
Supplement: Supplementary file 1 — Supplementary Information [file 41467_2020_14656_MOESM1_ESM.pdf]

Supplementary Information for

Glass Transition Temperature Correlation with Chemical Structure for Conjugated Polymers

Renxuan Xie[1], Albree R. Weisen[1], Youngmin Lee[1], Melissa A. Aplan[1], Abigail M. Fenton[1], Ashley E. Masucci[1], Fabian Kempe[2], Michael Sommer[2], Christian W. Pester[1], Ralph H. Colby[3,4], Enrique D. Gomez[1,3,4]**

[1]Department of Chemical Engineering, The Pennsylvania State University, University Park, PA 16802, USA

[2]Institut for Chemistry, Chemnitz University of Technology, Strasse der Nationen 62, 09111 Chemnitz, Germany.

[3]Department of Materials Science and Engineering, The Pennsylvania State University, University Park, PA 16802, USA

[4]The Materials Research Institute, The Pennsylvania State University, University Park, PA 16802, USA

*Correspondence to: rhc@plmsc.psu.edu, edg12@psu.edu

Supplementary Note 1

Chemical names and sources of 32 polymers examined in this work

PFTBT: poly((9,9-dioctylfluorene)-2,7-diyl-alt-[4,7-bis(thiophen-5-yl)-2,1,3-benzothiadiazole]-2′,2″-diyl). Synthesized.

PCDTBT: poly[N-9′-heptadecanyl-2,7-carbazole-alt-5,5-(4′,7′-di-2-thienyl-2′,1′,3′-benzothiadiazole)]. Synthesized.

PFT6BT: poly-((9,9-dioctylfluorene)-2,7-diyl-alt-[4,7-bis(3-hexylthiophen-5-yl)-2,1,3-benzothiadiazole]-2',2"-diyl). Synthesized.

PCT6BT: poly-((9-(9-heptadecanyl)-9H-carbazole)-1,4-diyl-alt-[4,7-bis(3-hexylthiophen-5-yl)-2,1,3-benzothiadiazole]-2',2"-diyl). Synthesized.

PDPT6BT: poly-(4-(2-ethylhexyl)-4H-dithieno[3,2-b:2',3'-d]pyrrole-2,6-diyl-alt-[4,7-bis(3-hexylthiophen-5-yl)-2,1,3-benzothiadiazole]-2’,2"-diyl)). Synthesized.

PF8BT: poly(9,9-dioctylfluorene-alt-benzothiadiazole). Purchased from Ossila.

PF8T2: poly(9,9-dioctylfluorene-alt-bithiophene). Purchased from Ossila.

TFB: poly(9,9-dioctylfluorene-co-N-(4-butylphenyl)diphenylamine). Data obtained from Liu et. al.[1]

APFO-Green9: Structure shown in Figure 1. Data obtained from Kroon et. al.[2]

APFO-18: Structure shown in Figure 1. Data obtained from Kroon et. al.[2]

PBTTT-C14 HH: poly[2,5-bis(3-tetradecylthiophen-2-yl)thieno[3,2-b]thiophene] with head-to-head configuration. Synthesized.

PT: poly(3-thiophene-2,5-diyl). Purchased from Sigma Aldrich.

P3BT: poly(3-butylthiophene-2,5-diyl). Purchased from Sigma Aldrich.

P3HT: poly(3-hexylthiophene-2,5-diyl). Purchased from Sigma Aldrich.

P3OT: poly(3-octylthiophene-2,5-diyl). Purchased from Sigma Aldrich.

P3DT: poly(3-decylthiophene-2,5-diyl). Purchased from Sigma Aldrich.

P3DDT: poly(3-dodecylthiophene-2,5-diyl). Purchased from Sigma Aldrich.

PF6: poly(9,9-di-n-hexylfluorenyl-2,7-diyl). Purchased from Lumtec.

PF2/6: poly[9,9-bis-(2-ethylhexyl)-9H-fluorene-2,7-diyl]. Data obtained from Papadopoulos et. al.[3]

PF8: poly(9,9-di-n-octylfluorenyl-2,7-diyl). Purchased from Ossila.

PF12: poly(9,9-di-n-dodecylfluorenyl-2,7-diyl). Purchased from Lumtec.

PffBT4T-2OD: poly[(5,6-difluoro-2,1,3-benzothiadiazol-4,7-diyl)-alt-(3,3’’’-di(2-octyldodecyl)-2,2’,5’,2’’,5’’,2’’’-quaterthiophen-5,5’’’-diyl)]. Purchased from 1-Material.

PTB7: poly[[4,8-bis[(2-ethylhexyl)oxy]benzo[1,2-b:4,5-b']dithiophene-2,6-diyl][3-fluoro-2-[(2-ethylhexyl)carbonyl]thieno[3,4-b]thiophenediyl]]. Purchased from Solarmer.

P(NDI2OD-T2): poly([N,N'-bis(2-octyldodecyl)naphthalene-1,4,5,8-bis(dicarboximide)-2,6-diyl]-*alt*-5,5'-(2,2'-bithiophene)). Purchased from Polyera.

PII-2T: poly(isoindigo-dithiophene). Synthesized.

P*mmp*P-4: polyarylene or poly(meta, meta, para-phenylene) with butyloxy side chains. Synthesized.

P*mmp*P-6: polyarylene or poly(meta, meta, para-phenylene) with hexyloxy side chains. Synthesized.

P*mmp*P-8: polyarylene or poly(meta, meta, para-phenylene) with octyloxy side chains. Synthesized.

P*mmp*P-10: polyarylene or poly(meta, meta, para-phenylene) with decyloxy side chains. Synthesized.

M3EH-PPV: poly[2,5-dimethoxy-1,4-phenylene-1,2-ethenylene-2-methoxy-5-(2-ethylhexyloxy)-1,4-phenylene-1,2-ethenylene]. Data obtained from Steffen et. al.[4]

MDMO-PPV: poly[2-methoxy-5-(3′,7′-dimethyloctyloxy)-1,4-phenylenevinylene]. Data obtained from Kim et. al.[5]

MEH-PPV: poly[2-methoxy-5-(2-ethylhexyloxy)-1,4-phenylene-1,2-ethenylene]. Data obtained from Kim et. al.[5]

Supplementary Note 2

Glass transition measurements of conjugated polymers

The glass transition temperature (T_g) in this work was measured under a nitrogen environment mostly by oscillatory shear rheometry as shown in Fig. 2 and Supplementary Figures 1-4. Exceptions include the amorphous polymers (PAr's) that show clear glass transition signatures using differential scanning calorimetry (DSC, Supplementary Figure 5), and the unmoldable polymer (PT) where DSC is the only option to probe its T_g (Supplementary Figure 6).

T_gs can be usually identified as the temperature that shows the local maximum of G'' and the decrease in G' starting from roughly 1 GPa (typical glassy modulus). But, some conjugated polymers exhibit two glass transition temperatures, one for the alkyl side chain and one for the backbone; the side chain T_g is the lower temperature transition because the side chains are more flexible than the backbone. The strength of the glass transition, represented by the peak magnitude of the loss modulus G'', is not necessarily higher for the backbone T_g than the that of the side chain, especially for highly crystalline conjugated polymers with branched side chains, as shown in Supplementary Figure 4. We speculate that the strength of the glass transition is controlled by the occupied volume of the segmental moiety that participates in the transition process. For conjugated polymers with high crystallinity and branched side chains, the T_g response of the side chain could be amplified because of the larger occupied volume of the branched side chain when compared to that of linear side chains, while the T_g response of the backbone could be diminished because of many backbone segments being fixed on a crystal lattice and not participating in the glass transition.

This behavior is demonstrated by comparing the T_g for PII-2T, PffBT4T-2OD, and P(NDI2OD)-T2, all of which share the same two branched octyl-decyl side chains (2OD) per repeat unit but have drastically different backbone structures. As shown in Supplementary Figure 4, their side chain T_gs vary between -50 and -60 °C and seem more apparent than their backbone T_gs, which are 65 °C for PII-2T, 74 °C for P(NDI2OD)-T2 and 28 °C for PffBT4T-2OD. PTB7, which has shorter branched side chains (ethyl-butyl) than 2OD and thus a lower side chain T_g at -80 °C, is also added to Supplementary Figure 4 for comparison. The backbone T_gs of these branched

semicrystalline conjugated polymers are identified by fitting the Gaussian peaks to the temperature dependence of the loss modulus.

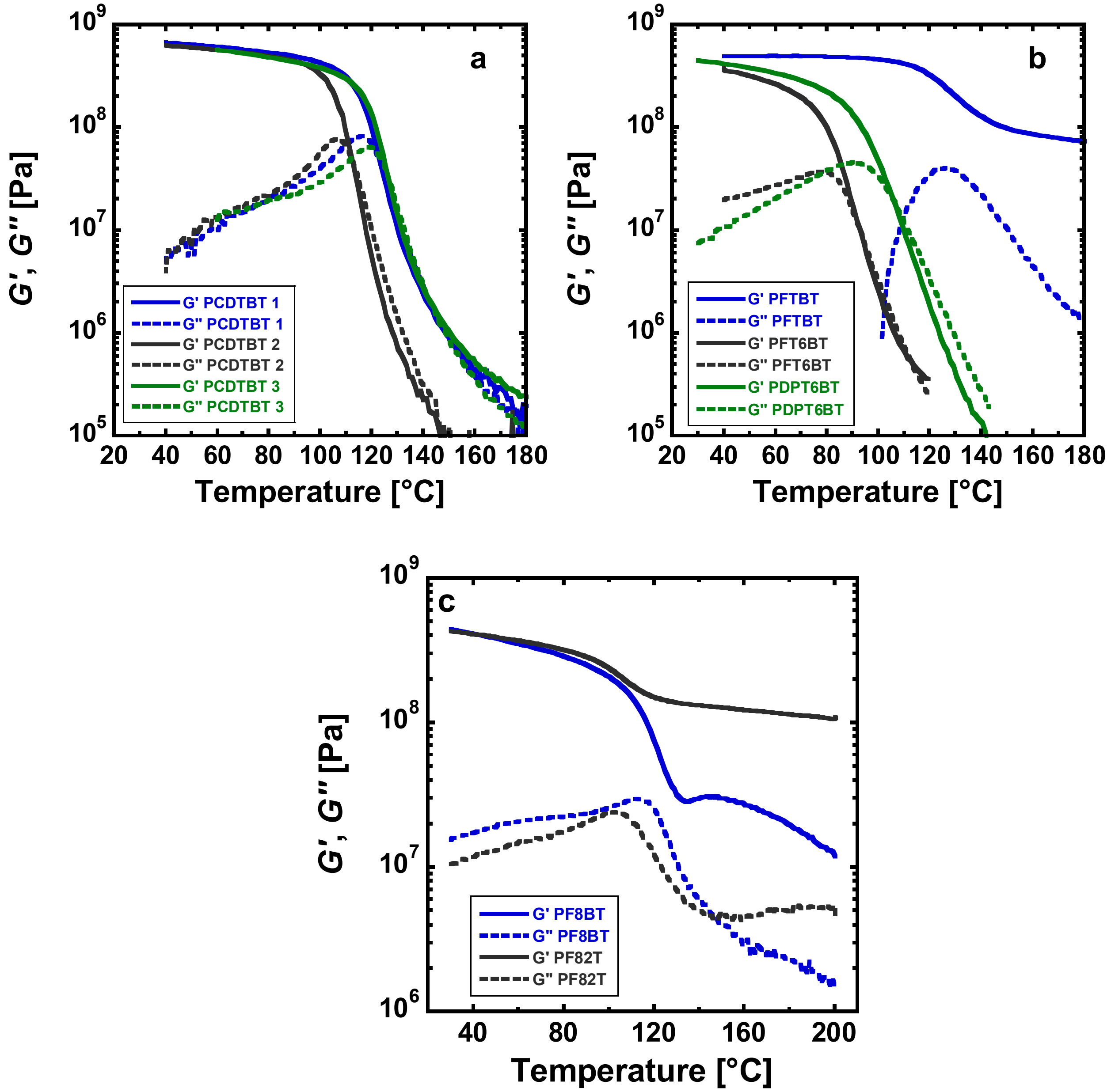

Supplementary Figure 1. T_g measurements of the push-pull conjugated polymers. Results shown for **a** three molecular weights of PCDTBT (107 °C, 116 °C, 119 °C), **b** PFTBT (126 °C), PFT6BT (79 °C) and PDPT6BT (90 °C), and **c** PF8BT (112 °C) and PF82T (102 °C). Oscillatory frequency of 1 rad/s, 5 °C/min heating and strain amplitude of 0.001.

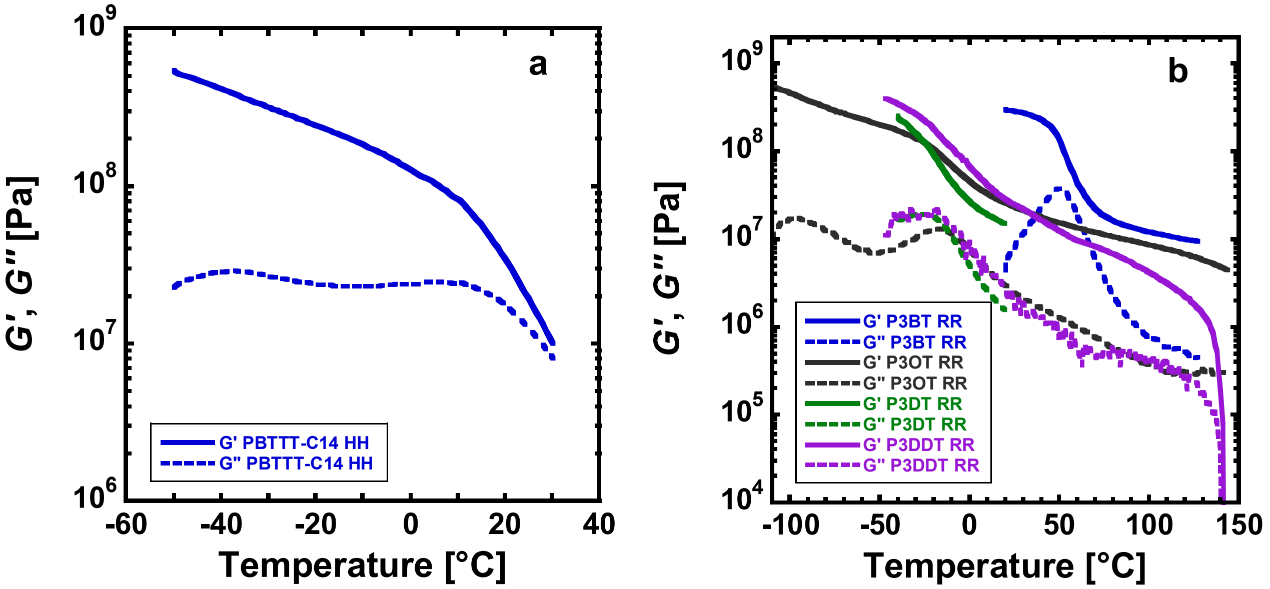

Supplementary Figure 2. T_g measurements of polythiophenes. Results shown for **a** PBTTT-C14 HH (5 °C) with head-to-head thiophenes, **b** regioregular P3BT (50 °C), P3OT (-17 °C), P3DT (-27 °C) and P3DDT (-18 °C). Oscillatory frequency of 1 rad/s, 5 °C/min heating and strain amplitude of 0.001.

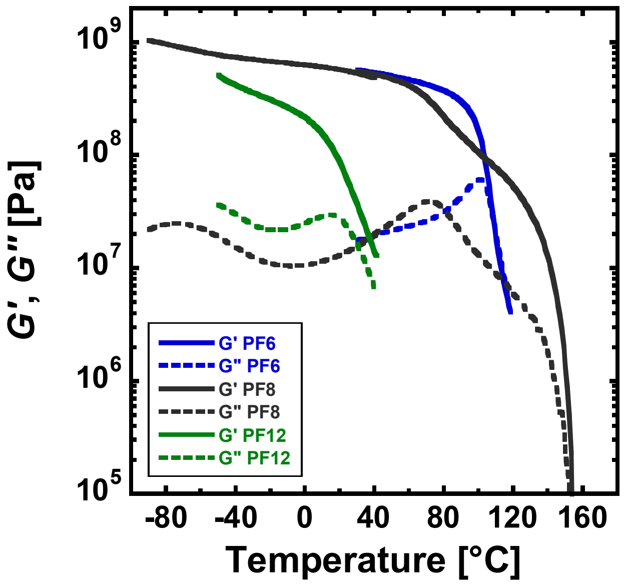

Supplementary Figure 3. T_g measurements of polyfluorenes, namely PF6 (101 °C), PF8 (71 °C) and PF12 (16 °C) with hexyl, octyl, and dodecyl side chains, respectively. Oscillatory frequency of 1 rad/s, 5 °C/min heating and strain amplitude of 0.001.

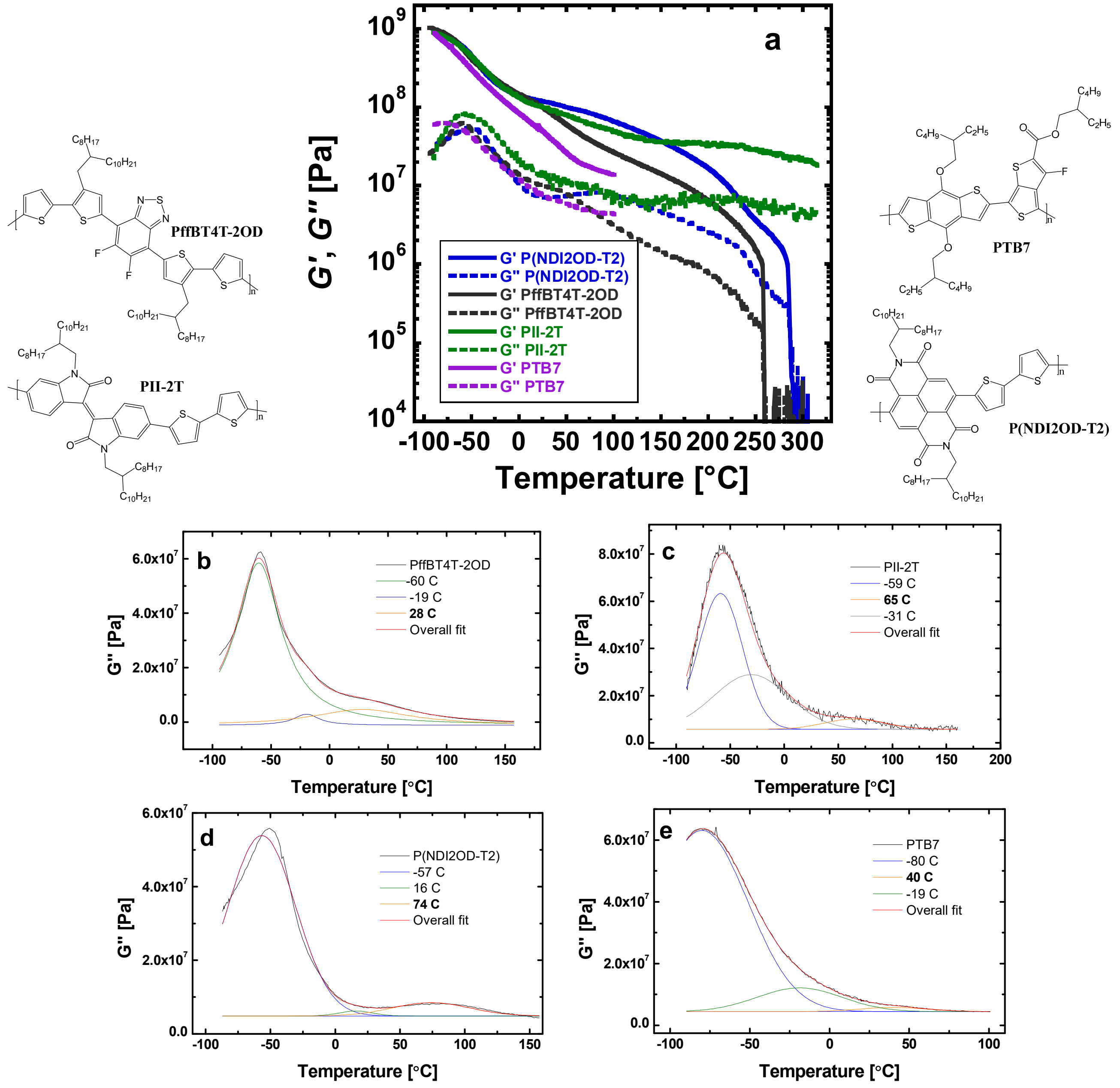

Supplementary Figure 4. Rheology identifies T_gs of conjugated polymers. **a** T_g measurements by rheology for semicrystalline conjugated polymers with branched alkyl side chains, including PII-2T, PffBT4T-2OD, P(NDI2OD-T2) and PTB7, conducted at a heating rate of 5 °C/min, an oscillatory frequency of 1 rad/s and a strain amplitude of 0.001. The side chain T_g is more apparent and at a lower temperature than the backbone T_g. The data for PTB7 stop at 100 °C, above which the glue (polycyanoacrylate, only used for PTB7) between the plate and sample softens and the data no longer represent the modulus of the sample. The extraction of backbone T_gs of **b** PffBT4T-2OD, **c** PII-2T, **d** P(NDI2OD-T2), **e** PTB7 are shown by fitting the temperature dependence of the loss modulus with Gaussian peaks. The peak temperature that corresponds to each Gaussian fit is shown in the legend, and the backbone T_gs are marked in bold. Chemical structures of these low bandgap polymers with branched side chains are also shown.

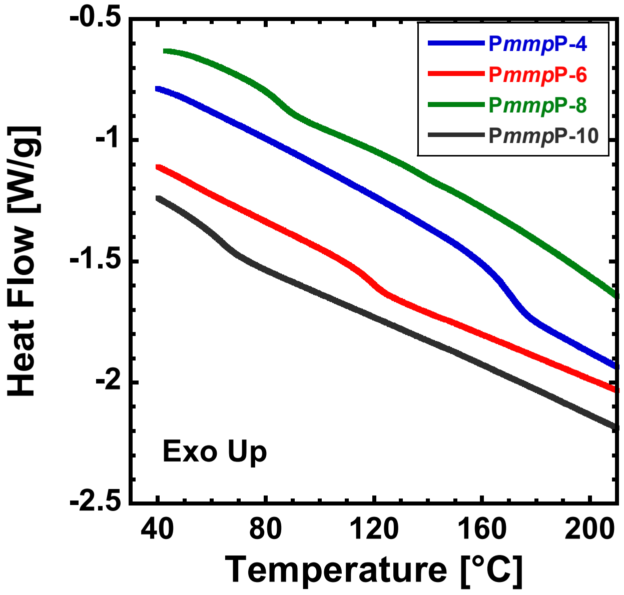

Supplementary Figure 5. Second DSC heating scans of P*mmp*P-4, P*mmp*P-6, P*mmp*P-8, P*mmp*P-10 at 10 °C/min. T_gs are identified as the midpoints of the step changes in heat flow: 164 °C for P*mmp*P-4, 117 °C for P*mmp*P-6, 87 °C for P*mmp*P-8, 58 °C for P*mmp*P-10.

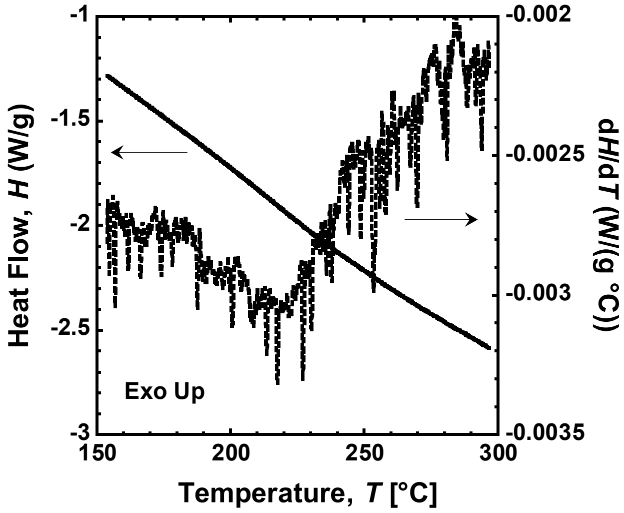

Supplementary Figure 6. Second DSC heating scan of the unsubstituted polythiophene (PT) powder at 10 °C/min. T_g of PT is identified at 215 °C where a small step change in heat flow is apparent (more clearly seen in the derivative).

Supplementary Note 3

Summary of T_gs and other parameters used in this work

Supplementary Table 1 summarizes glass transition temperatures used in Figure 3 and Figure 4 of the main text. Effective mobility values (ζ) and side chain mass fraction (w) are calculated for all studied polymers based on the repeat unit structure. The mass density (ρ) is estimated by conforming the sample into a cylinder between two (3 ± 0.1)-mm parallel plates, while the density of the unsubstituted polythiophene (PT) is calculated to be 1.55 g/ml based on the crystal unit cell[6]. The persistence length (l_p) is estimated by the Freely Rotating Model[7] when experimental values from small-angle neutron scattering (SANS)[8] or static light scattering (SLS)[9, 10] are not available in the literature. Packing length is calculated from the estimated l_p and ρ according to Supplementary Equation (1) and summarized in Supplementary Table 1.

Supplementary Table 1. Summary of the experimentally measured backbone glass transition temperature (T_g), predicted T_g by Equation (4), determination method, effective mobility value (ζ), side chain mass fraction (w), mass density at 298 K (ρ), persistence length (l_p), number of Kuhn monomers (N_k), number average molecular weight (M_n), and packing length (p) of conjugated polymers examined in this work.

Group	Polymer	M_n [kDa]	Method	Exp. T_g [°C]	Pred. T_g [°C]	ζ [-]	w [-]	ρ [g/ml]	l_p [nm]	N_k [-]	p [Å]
1	P3HT RR	14.5*	rheo	1*	18	0.873	0.515	1.1	2.7	6.3	1.17
		15.3*	rheo	3*	18	0.873	0.515	1.1	2.7	6.6	1.17
		24.2*	rheo	10*	18	0.873	0.515	1.1	2.7	11	1.17
		21.5*	rheo	10*	18	0.873	0.515	1.1	2.7	9.3	1.17
		27.7*	rheo	11*	18	0.873	0.515	1.1	2.7	12	1.17
		36.6*	rheo	14*	18	0.873	0.515	1.1	2.7	16	1.17
	P3HT RRa	41.7*	rheo	4*	18	0.873	0.515	1.1	2.7	18	1.17
		44.9*	rheo	6*	18	0.873	0.515	1.1	2.7	20	1.17
	P3DDT RR	44.7	rheo	-18	-32	0.918	0.670	0.99	2.7	13	1.96
	P3OT RR	22.6	rheo	-17	-4	0.892	0.580	1.03	2.7	8.4	1.46
	P3BT RR	35.0	rheo	50	49	0.844	0.410	1.15	2.7	18	0.930
	P3DDT RRa	46.4	rheo	-30	-32	0.918	0.670	0.99	2.7	13	1.96
	P3DT RR	47.7	rheo	-27	-20	0.907	0.635	-	2.7	16	1.72[j]
	P3OT RRa	22.6	rheo	-19	-4	0.892	0.580	1.03	2.7	8.4	1.46
	P3BT RRa	29.2	rheo	45	49	0.844	0.410	1.15	2.7	15	0.930
	PBTTT-C14 HH	16.8	rheo	5	-8	0.896	0.560	1.05	4.1	4.1	0.997
	PffBT4T-2OD	12.9	rheo	28	11	0.879	0.541	-	3.7	3.7	1.04[j]
	PTB7	19.8	rheo	40	27	0.864	0.549	-	8.7	1.9	0.562[j]
	PDPT6BT	3.5	rheo	90	76	0.820	0.374	1.25	4.2	1.1	0.622
	PT	-	DSC	215	186	0.720	0.000	1.55 [k]	2.7	-	0.410

**2**	PFTBT	16.0 [*]	rheo	132 [*]	121	0.779	0.329	1.35	5.5	4.4	0.369
		11.7 [*]	rheo	126 [*]	121	0.779	0.329	1.35	5.5	3.2	0.369
		9.3 [*]	rheo	122 [*]	121	0.779	0.329	1.35	5.5	2.6	0.369
	PCDTBT	6.7 [^]	rheo	107	125	0.776	0.340	1.30	5.9	1.6	0.381
		7.6 [^]	rheo	116	125	0.776	0.340	1.30	5.9	1.9	0.381
		15 [^]	rheo	119	125	0.776	0.340	1.30	5.9	3.7	0.381
	PCT6BT	21.6	rheo	74	76	0.820	0.471	1.15	5.9	4.3	0.535
	PFT6BT	34.7	rheo	79	72	0.823	0.464	1.15	5.5	7.7	0.540
	PF6	44	rheo	101	89	0.808	0.512	1.10	7.0 [g]	8.2	0.413
	PF8	31	rheo	71	60	0.834	0.582	1.03	7.0 [g]	4.9	0.516
	PF2/6	98 [a]	DSC	59 [a]	60	0.834	0.582	-	7.0 [g]	16	0.516 [j]
	PF12	33.5	rheo	16	20	0.870	0.676	0.99	7.0 [g]	4.1	0.692
	PF82T	21.4	rheo	102	92	0.805	0.409	1.20	4.9	6.5	0.477
	PF8BT	88	rheo	112	103	0.795	0.433	1.15	10 [h]	11	0.294
	P*mmp*P-4	27.7	DSC	164	161	0.743	0.306	-	0.9	45	3.03 [j]
	P*mmp*P-6	67.0	DSC	117	125	0.775	0.397	-	0.9	94	3.49 [j]
	P*mmp*P-8	46.5	DSC	87	98	0.800	0.467	-	0.9	58	3.95 [j]
	P*mmp*P-10	48.7	DSC	58	76	0.820	0.567	0.99	0.9	54	4.45
	P(NDI2OD-T2)	109	rheo	74	47	0.846	0.568	-	3.4	25	1.54 [j]
	PII-2T	12.1 [b]	rheo	65	47	0.846	0.577	-	6.2	1.9	0.684 [j]
	M3EH-PPV	> 20 [c]	DSC	113 [c]	105	0.794	0.526	-	6.0 [i]	5.2	0.441 [j]
	MEH-PPV	45 [d]	DSC	66 [d]	63	0.832	0.615	-	6.0 [i]	9.6	0.543 [j]
	MDMO-PPV	123 [d]	DSC	50 [d]	45	0.848	0.653	-	6.0 [i]	24	0.601 [j]
	TFB	57 [e]	DSC	140 [e]	140	0.762	0.412	-	2.4	29	1.36 [j]
	APFO-18	8 [f]	DSC	142 [f]	173	0.731	0.272	-	5.5	1.8	0.603 [j]
	APFO-Green9	10 [f]	DSC	192 [f]	204	0.704	0.230	-	5.5	1.9	0.715 [j]

[*]: T_gs and M_n determined from our previous work.[11]

[^]: M_ns for PCDTBTs determined by GPC in 1,2,4-trichlorobenzene at 150 °C using universal calibration.

[a]: T_g and M_n for PF2/6 from Papadopoulos et. al.[3]

[b]: M_n for PII-2T determined by GPC in 1,2,4-trichlorobenzene at 160 °C using calibration relative to polystyrene from Zhou et. al. [12]

[c]: T_g and M_n for M3EH-PPV from Steffen et. al. [4]

[d]: T_gs and M_n for MEH-PPV and MDMO-PPV from Kim et. al.[5]

[e]: T_g and M_n for TFB from Liu et. al.[1]

[f]: T_gs and M_n for APFO-18 and APFO-Green9 from Kroon et. al.[2]

[g]: l_p of PFs determined by SANS to be 7.0 nm.[8]

[h]: l_p of PF8BT determined by SLS to be 10.0 nm.[9]

[i]: l_p of PPVs with alkyl side chains determined by SLS to be 6.0 nm.[10]

[j]: p is computed by Supplementary Equation (1) assuming density of 1.0 g/ml.

[k]: ρ for PT calculated from the crystal unit cell. [6]

All other T_gs are measured in this work.

All other l_ps are predicted by freely rotating chain model in this work.

All other ρs are obtain by measuring the volume using a parallel plate geometry with a well-defined gap for samples of known mass.

All other M_ns are determined by GPC in chlorobenzene at 40 °C using a polystyrene-relative calibration.

All N_ks are determined from the tabulated M_n and l_p and the repeat unit length, l_0. (*i.e.*, $N_k = M_n/(2l_pM_0/l_0)$)

Supplementary Note 4

Attempt to correlate packing length with T_g

The packing length (p) depends on monomer volume and chain stiffness, so we attempted to correlate p with the glass transition temperatures of conjugated polymers. The persistence length (l_p) is used to quantify the chain stiffness as the length of chain over which the chain orientation remains correlated. The Kuhn monomer volume (v_0) represents the volume of monomers within a Kuhn length and is strongly affected by the alkyl side chain length. The packing length (p) is then defined as the ratio of Kuhn monomer volume (v_0) over the square of the Kuhn length ($l_k = 2l_p$),[13] and can be calculated from l_p, the molar mass of repeat unit (M_0), the mass density (ρ) and the repeat unit length (l_0) using

$$p = \frac{v_0}{(2l_p)^2} = \frac{M_0}{2\rho l_0 l_p} \tag{1}$$

Experimentally measured l_ps are available for several conjugated polymers in the literature from either static light scattering (SLS) or small angle neutron scattering (SANS), such as 2.8 nm for P3HT[14, 15], 7.0 nm for PF-EH[8], 10.0 nm for PF8BT[9], and 6.0 nm for PPV with alkyl side chains[10]. Alternatively, we can predict the persistence length using the freely rotating chain (FRC) model.[7] This model is validated by the close match of the predicted l_p for P3HT (2.7 nm) versus results by SANS (3.0 nm)[14]. This generalized freely rotating model[7] handles different bond lengths and angles within the repeat unit. Specifically, by first constructing the repeat unit in Avogadro, the lengths of rigid segments and deflection angles are measured after minimizing the energy of the molecule using a force field (MMFF94s) and the steepest descent algorithm.[16] Then, by allowing each rigid segment to freely rotate about its deflection angle, the tangent-tangent correlation as a function of the number of repeat units is computed by numerically averaging many different possible conformations (50,000 in this case). l_p (which is $l_0 N_p$) is then the characteristic length of this exponentially-decaying tangent-tangent correlation function:

$$\langle v_0 \cdot v_n \rangle = \exp\left(-\frac{n}{N_p}\right) \tag{2}$$

where v_o and v_n are backbone tangents, n is the number of repeat units, and N_p is the number of repeat units in a persistence length. Examples of the tangent-tangent correlation function for PBTTT-C14 and PFTBT are shown in Supplementary Figure 7.

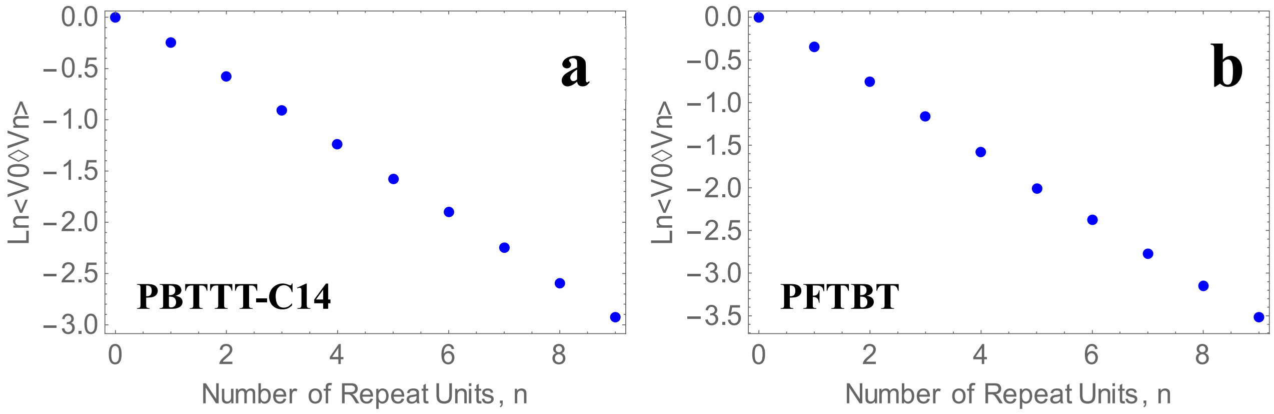

Supplementary Figure 7. Extracting the persistence length ($l_p = N_p \times l_0$) from the exponentially decaying tangent-tangent correlation obtained from the freely rotating chain model. Results for **a** PBTTT-C14 (l_p = 2.98 × 1.36 nm = 4.1 nm) and **b** PFTBT (l_p = 2.65 × 2.08 nm = 5.5 nm).

Using the l_p (obtained by either experiment or FRC) and the estimated mass density, the packing length for conjugated polymers is calculated according to Supplementary Equation (1) and summarized in Supplementary Table 1. Although the correlation between T_g and the packing length (p) works well for a range of relatively stiff conjugated polymers (*e.g.*, polythiophenes, polyfluorenes, and push-pull polymers) in Supplementary Figure 8, it completely fails to describe polyarylenes (PAr) and TFB, both of which have much smaller persistence lengths due to their kinked backbone structures. As for P(NDI2OD-T2), its Kuhn monomer volume might scale differently with the persistence length, due to its large two-dimensional naphthalene-carboximide unit.

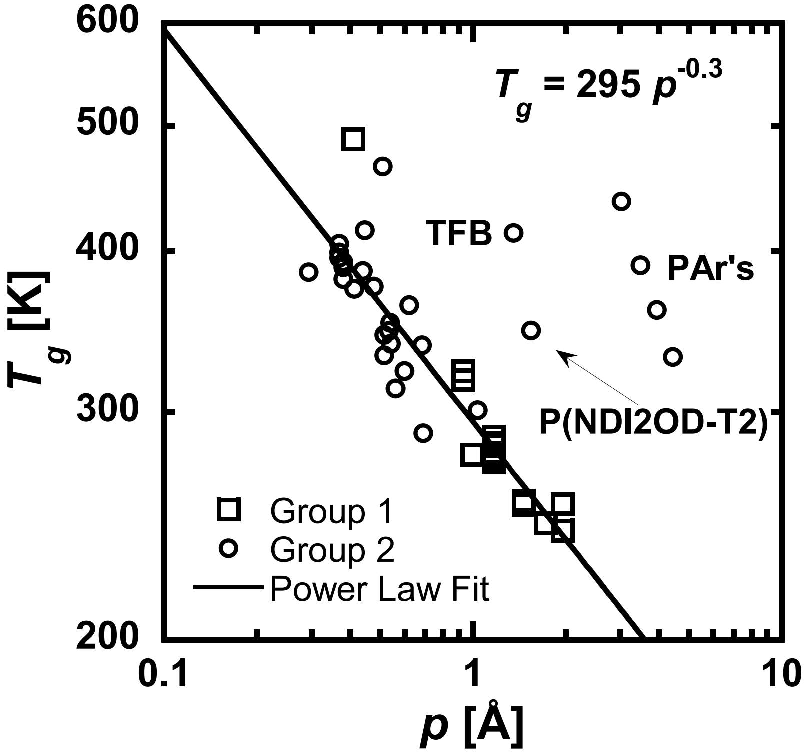

Supplementary Figure 8. Correlation between the packing length (*p*) and the glass transition temperature (T_g) on log-log scale for conjugated polymers with alkyl side chains. Outliers are polyarylenes (PAr's), TFB and P(NDI2OD-T2). The solid curve is a power law fit through most of the conjugated polymers.

Supplementary Note 5

The Strength of Correlation between T_g and Effective Mobility Value

As discussed in the main text, there is only one adjustable parameter in our model. This parameter determines the effective mobility of the repeat unit, namely the relative atomic mobility between a conjugated atom and a non-conjugated atom. The correlation between the glass transition temperature (T_g) and the effective mobility value (ζ) is the strongest (i.e. $R^2 = 0.956$ in Fig. 4a) when the atomic mobility value in a thiophene ring follows $1.2a = 0.72$ (*i.e.*, $a = 0.6$), or equivalently the mobility per ring is $0.72 \times 5 = 3.6$. As shown in Supplementary Figure 9, the strength of this correlation decreases as a deviates from 0.6 and eventually becomes poor (i.e. $R^2 < 0.91$) for a greater than 0.68 or less than 0.45.

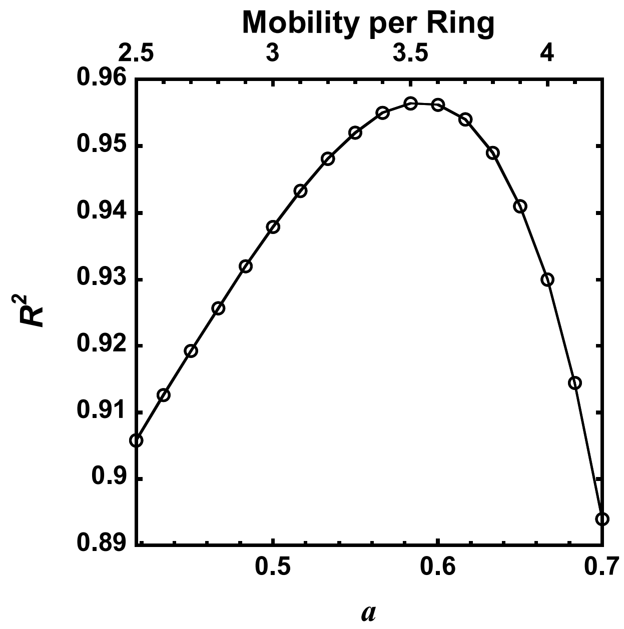

Supplementary Figure 9. The strength of correlation represented by the coefficient of determination (R^2) between T_g and ζ depends on a. The strongest correlation is achieved when the mobility per ring is 3.6 or equivalently $a = 0.6$, while the correlation becomes poor if atoms in a conjugated ring are assigned to be too immobile ($a < 0.45$) or too mobile ($a > 0.68$) relative to the atoms on the side chain.

Supplementary Note 6

Polymers with Aromatic Groups on Side Chains

Currently, the general correlation between T_g and ζ is established using conjugated polymers with alkyl side chains. But, for polymers with a phenyl ring attached to the free end of a side chain, a clear deviation from the correlation is expected due to the different atomic mobilities than that of pure alkyl side chains. Specifically, as shown in Supplementary Figure 10a, the values of ζ for both polystyrene (PS) and poly[2-methoxy-5-(2′-phenylethoxy)-1,4-phenylenevinylene] (MPE-PPV) are underestimated based on the original assignment of atomic mobility in Table 2, since a phenyl ring on a side chain is in fact a lot more mobile than that on a backbone. Increasing the atomic mobility value for an atom in a phenyl ring ($\zeta_{\text{phenyl, side chain}}$) from 0.60 to 0.72, both PS and MDE-PPV agree with the correlation again in Supplementary Figure 10b. Note that the T_g of PS is 100 °C[17], while T_g of MPE-PPV is 111 °C[18].

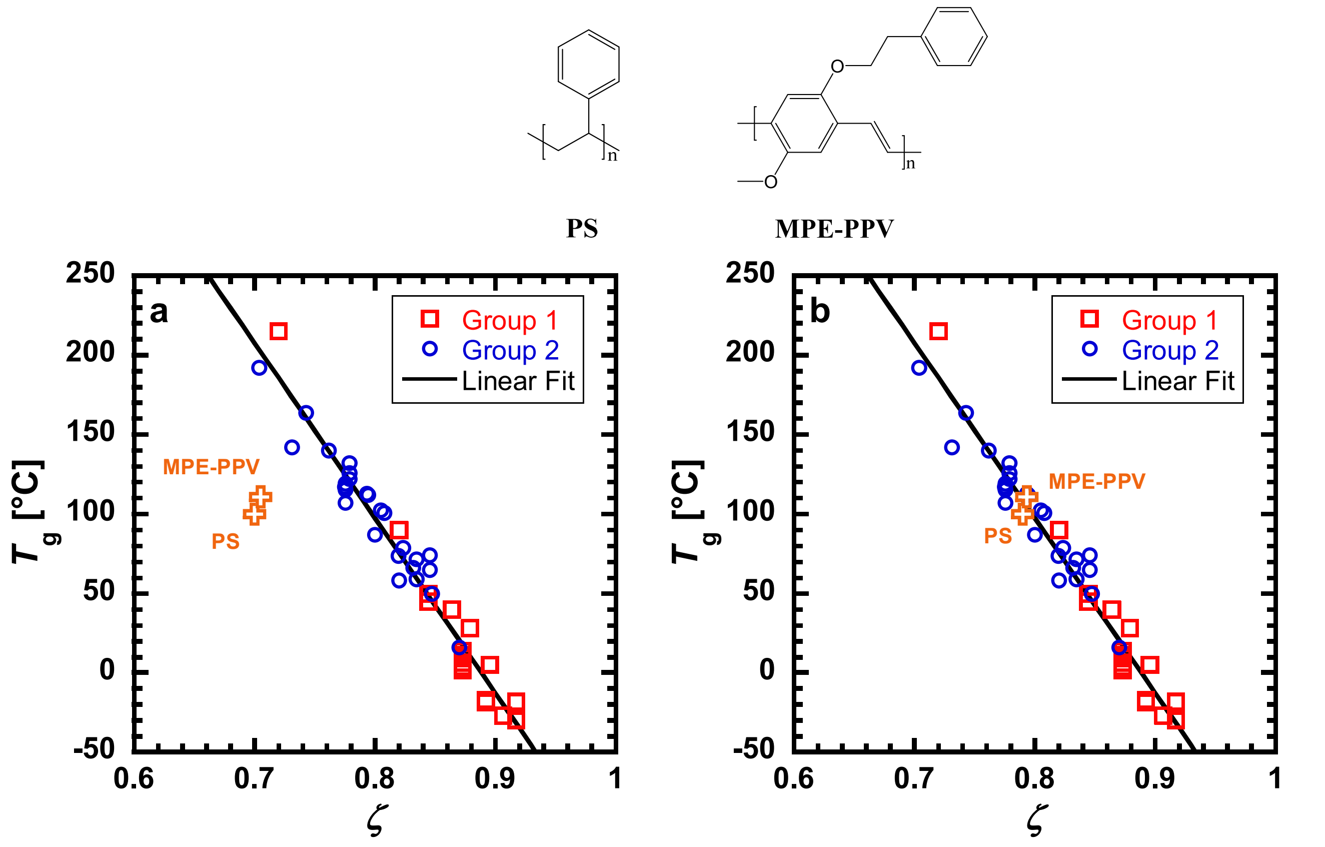

Supplementary Figure 10. Incorporating polymers with aromatic groups on the side chain in the model. a. Data for MPE-PPV and PS lie far below the correlation based on the original assignment of the atomic mobility values in Table 2. **b** Data for MPE-PPV and PS agree with the correlation after increasing the atomic mobility values in phenyl rings on side chains from 0.60 to 0.72. Chemical structures of PS and MPE-PPV are also shown.

Supplementary Note 7

Molecular weight characterization

As shown in Supplementary Table 1, the M_ns were determined directly by the gel permeation chromatography (GPC) relative to polystyrene standards for the following samples, P3OT RR, P3BT RR, P3DDT RRa, P3DT RR, P3OT RRa, P3BT RRa, PBTTT-C14 HH, PffBT4T-2OD, PTB7, PDPT6BT, PCT6BT, PFT6BT, PF6, PF8, PF12, PF82T, PF8BT, PmmpP-4, PmmpP-6, PmmpP-8, PmmpP-10, and P(NDI2OD-T2). GPC was run on an Agilent Technologies 1260 Infinity Series GPC/SEC. Samples were characterized with a 0.50 ml/min flow rate at 40 °C in chlorobenzene. Relative calibration was done using polystyrene standards from Agilent Technologies that range from 364.00 kDa to 0.37 kDa. Most samples were prepared at a concentration of 1.00 mg/ml, except for PffBT4T-2OD, PDPT6BT, and PF6 (0.50 mg/ml), P3BT RR (0.25 mg/ml), and P(NDI2OD-T2) (0.33 mg/ml). For other samples in Supplementary Table 1, the M_ns were either taken directly from the cited literatures or determined by other methods, such as ^{1}H-NMR, SLS and GPC using universal calibration.

Supplementary References

1. Liu, D.; Osuna Orozco, R.; Wang, T. Deviations of the glass transition temperature in amorphous conjugated polymer thin films. *Physical Review E* **2013,** 88 (2), 022601
2. Kroon, R. Synthesis and properties of pi-conjugated polymers for organic photovoltaics. Chalmers University of Technology, 2013.
3. Papadopoulos, P.; Floudas, G.; Chi, C.; Wegner, G. Molecular dynamics of oligofluorenes: A dielectric spectroscopy investigation. *The Journal of Chemical Physics* **2004,** 120 (5), 2368-2374
4. Steffen, P.; Hans-Heinrich, H. Investigation of poly(arylene vinylene)s, 41. Synthesis of soluble dialkoxy-substituted poly(phenylene alkenylidene)s by applying the Horner-reaction for condensation polymerization. *Macromolecular Chemistry and Physics* **1999,** 200 (8), 1870-1878
5. Kim, J. Y.; Frisbie, C. D. Correlation of Phase Behavior and Charge Transport in Conjugated Polymer/Fullerene Blends. *J. Phys. Chem. C* **2008,** 112 (45), 17726-17736
6. Mo, Z.; Lee, K. B.; Moon, Y. B.; Kobayashi, M.; Heeger, A. J.; Wudl, F. X-ray scattering from poly(thiophene): crystallinity and crystallographic structure. *Macromolecules* **1985,** 18 (10), 1972-1977
7. Zhang, W.; Gomez, E. D.; Milner, S. T. Predicting Chain Dimensions of Semiflexible Polymers from Dihedral Potentials. *Macromolecules* **2014,** 47 (18), 6453-6461
8. Fytas, G.; Nothofer, H. G.; Scherf, U.; Vlassopoulos, D.; Meier, G. Structure and Dynamics of Nondilute Polyfluorene Solutions. *Macromolecules* **2002,** 35 (2), 481-488
9. Grell, M.; Bradley, D. D. C.; Long, X.; Chamberlain, T.; Inbasekaran, M.; Woo, E. P.; Soliman, M. Chain geometry, solution aggregation and enhanced dichroism in the liquidcrystalline conjugated polymer poly(9,9-dioctylfluorene). *Acta Polymerica* **1998,** 49 (8), 439-444
10. Gettinger, C. L.; Heeger, A. J.; Drake, J. M.; Pine, D. J. A photoluminescence study of poly(phenylene vinylene) derivatives: The effect of intrinsic persistence length. *The Journal of Chemical Physics* **1994,** 101 (2), 1673-1678
11. Xie, R.; Lee, Y.; Aplan, M. P.; Caggiano, N. J.; Müller, C.; Colby, R. H.; Gomez, E. D. Glass Transition Temperature of Conjugated Polymers by Oscillatory Shear Rheometry. *Macromolecules* **2017,** 50 (13), 5146–5154
12. Zhou, Y.; Kurosawa, T.; Ma, W.; Guo, Y.; Fang, L.; Vandewal, K.; Diao, Y.; Wang, C.; Yan, Q.; Reinspach, J.; Mei, J.; Appleton, A. L.; Koleilat, G. I.; Gao, Y.; Mannsfeld, S. C. B.; Salleo, A.; Ade, H.; Zhao, D.; Bao, Z. High Performance All-Polymer Solar Cell via Polymer Side-Chain Engineering. *Advanced Materials* **2014,** 26 (22), 3767-3772
13. Witten, T. A.; Milner, S. T.; Wang, Z.-G., *Theory of Stress Distribution in Block Copolymer Microdomains*. Plenum Press: New York, 1989; p 659.
14. McCulloch, B.; Ho, V.; Hoarfrost, M.; Stanley, C.; Do, C.; Heller, W. T.; Segalman, R. A. Polymer Chain Shape of Poly(3-alkylthiophenes) in Solution Using Small-Angle Neutron Scattering. *Macromolecules* **2013,** 46 (5), 1899-1907
15. Heffner, G. W.; Pearson, D. S. Molecular characterization of poly(3-hexylthiophene). *Macromolecules* **1991,** 24 (23), 6295-6299
16. Hanwell, M. D.; Curtis, D. E.; Lonie, D. C.; Vandermeersch, T.; Zurek, E.; Hutchison, G. R. Avogadro: an advanced semantic chemical editor, visualization, and analysis platform. *Journal of Cheminformatics* **2012,** 4 (1), 17
17. Mark, J. E., *Polymer data handbook*. 2nd ed.; Oxford University Press: Oxford ; New York, 2009; p vii, 1250 p.
18. Vandenbergh, J.; Conings, B.; Bertho, S.; Kesters, J.; Spoltore, D.; Esiner, S.; Zhao, J.; Van Assche, G.; Wienk, M. M.; Maes, W.; Lutsen, L.; Van Mele, B.; Janssen, R. A. J.; Manca, J.; Vanderzande, D. J. M. Thermal Stability of Poly[2-methoxy-5-(2′-phenylethoxy)-1,4-phenylenevinylene] (MPE-PPV):Fullerene Bulk Heterojunction Solar Cells. *Macromolecules* **2011,** 44 (21), 8470-8478
